# Supplementary material for: Exploring the impact of Brownian motion on novel closed-form solutions of the extended Kairat-II equation
Source: PLoS One. 2025 Jan 16;20(1):e0314849. doi: 10.1371/journal.pone.0314849 (PMC11737771; doi:10.1371/journal.pone.0314849)
Supplement: Attachment — Submitted filename: Review rep..docx [file pone.0314849.s001.docx]

The article explores the impact of Brownian motion on the extended version of the Kairat-II equation, which is a non-linear differential equation used to model wave dynamics. The primary objective is to incorporate stochastic behavior (random perturbations) into the deterministic equation and derive closed-form analytical solutions using two distinct methods—the modified Tanh method (linked with the Riccati equation) and the Sardar sub-equation method (SSEM). These solutions are analyzed to understand the influence of noise intensity on wave behavior, which has implications for modeling real-world wave dynamics, such as in plasma physics, optical communication, and marine environments.

Overall, the article is well written and justifies its impact for possible publication in PLoS ONE provided the following observations are responded positively:

1.	Although several types of solutions (e.g., bright, dark, and singular solitons) are presented, the article lacks details related to physical interpretations of these solutions in real-world systems, such as plasma or marine environments. A more explicit connection between the solutions and their potential physical implications would enhance the work's impact.

2.	The study observes that high-energy regions are more affected by noise, but it lack the reasons behind this happening.

3.	The literature review part can be improved, particularly, by discussing exact solutions, soliton dynamics, and algebraic properties related to non-linear and fractional differential equations, which align closely with the objectives of current study. To enhance the theoretical foundation of the research and to demonstrate the broader relevance of the findings in the field of non-linear wave analysis and stochastic integrable systems, I would like to suggest the inclusion of the following recent studies:

Kai, Y., & Yin, Z. (2021). On the Gaussian traveling wave solution to a special kind of Schrödinger equation with logarithmic nonlinearity. Modern Physics Letters B, 36(02), 2150543. https://doi.org/10.1142/S0217984921505436

Kai, Y., Chen, S., Zhang, K., & Yin, Z. (2022). Exact solutions and dynamic properties of a nonlinear fourth-order time-fractional partial differential equation. Waves in Random and Complex Media. https://doi.org/10.1080/17455030.2022.2044541

Zhu, C., Al-Dossari, M., Rezapour, S., & Gunay, B. (2024). On the exact soliton solutions and different wave structures to the (2+1) dimensional Chaffee–Infante equation. Results in Physics, 57, 107431. https://doi.org/10.1016/j.rinp.2024.107431

Guo, S., & Wang, S. (2024). Twisted relative Rota-Baxter operators on Leibniz conformal algebras. Communications in Algebra, 52(9), 3946-3959. https://doi.org/10.1080/00927872.2024.2337276.
