# Supplementary material for: Exploring the impact of Brownian motion on novel closed-form solutions of the extended Kairat-II equation
Source: PLoS One. 2025 Jan 16;20(1):e0314849. doi: 10.1371/journal.pone.0314849 (PMC11737771; doi:10.1371/journal.pone.0314849)
Supplement: Attachment — Submitted filename: Kairat-II model Response letter.docx [file pone.0314849.s002.docx]

Reviewer #1: The research article titled "Exploring the Impact of Brownian Motion on Novel Closed-Form Solutions of the Extended Kairat-II Equation" presents a valuable contribution to the study of stochastic systems by incorporating Brownian motion into the deterministic Kairat-II equation. The authors employ two mathematical approaches—the modified Tanh method and the Sardar Sub-Equation Method (SSEM)—to derive a variety of closed-form solutions. These solutions, expressed through trigonometric and hyperbolic functions, are visualized in 2D and 3D to demonstrate distinct wave behaviors, such as bright, dark, singular, and periodic solitons. The article provides interesting insights into how random perturbations affect wave dynamics, showing that high-energy regions are more vulnerable to noise than low-energy regions, which remain relatively robust.

The incorporation of Brownian motion into the Kairat-II equation through Ito calculus represents a novel approach to studying the dynamics of stochastic systems. This framework is promising for practical applications in plasma physics, optical communications, and marine environments, where random perturbations play a significant role. Additionally, the comparison of singular solutions generated by the modified Tanh method with the non-singular solutions obtained through the SSEM method provides a rich mathematical insight into the nature of the solutions.

While the article offers several strengths, some improvements in the literature review could enhance the study’s theoretical depth and relevance. In particular, the authors could strengthen their work by citing recent studies on nonlinear and stochastic differential equations.

Despite the article’s strengths, a few limitations are noted. The model assumes Brownian motion as the primary source of noise, but real-world systems often involve more complex noise types, such as colored noise or Lévy processes. Discussing the limitations of using Brownian motion and suggesting potential extensions to more complex stochastic environments would provide a more comprehensive outlook for future research. In addition, a comparative analysis with other stochastic systems, if possible, could contextualize the findings and highlight the novel aspects of the current study more effectively.
More so, while the use of advanced mathematical techniques like the modified Tanh method and SSEM is commendable, the article could benefit from providing step-by-step explanations or an appendix with detailed derivations. This would make the work more accessible to readers who may not be familiar with these specialized techniques.

**Response: We are really thankful for the positive comments and appreciation of our work we have tried our best to present clear and easily readable work. We have taken the above comments into consideration and added some recent works in this area for comparison. Besides this we have added that in future this work can be re-visited using a more complex noise and more importantly can use neural network to study some more novel behaviors in the proposed model. The method is straightforward presented that how we applied so due to the length of the article we omitted to present some more details.**

Reviewer #2: The article explores the impact of Brownian motion on the extended version of the Kairat-II equation, which is a non-linear differential equation used to model wave dynamics. The primary objective is to incorporate stochastic behavior (random perturbations) into the deterministic equation and derive closed-form analytical solutions using two distinct methods—the modified Tanh method (linked with the Riccati equation) and the Sardar sub-equation method (SSEM). These solutions are analyzed to understand the influence of noise intensity on wave behavior, which has implications for modeling real-world wave dynamics, such as in plasma physics, optical communication, and marine environments.
Overall, the article is well written and justifies its impact for possible publication in PLoS ONE provided the following observations are responded positively:
1. Although several types of solutions (e.g., bright, dark, and singular solitons) are presented, the article lacks details related to physical interpretations of these solutions in real-world systems, such as plasma or marine environments. A more explicit connection between the solutions and their potential physical implications would enhance the work's impact.

**Response: We have now elaborated on the potential physical implications of the derived solutions, particularly their relevance in plasma and marine environments. In plasma systems, the stability and structure of soliton solutions can represent energy transfer and wave propagation under perturbative influences. In marine environments, solitons reflect tidal and surface wave dynamics, which could be subject to noise, as seen in our stochastic model.**
2. The study observes that high-energy regions are more affected by noise, but it lack the reasons behind this happening.

**Response: We have expanded the discussion to include potential reasons why high-energy regions are more affected by noise, drawing from principles of wave dynamics and energy distribution in stochastic systems.**

3. The literature review part can be improved, particularly, by discussing exact solutions, soliton dynamics, and algebraic properties related to non-linear and fractional differential equations, which align closely with the objectives of current study. To enhance the theoretical foundation of the research and to demonstrate the broader relevance of the findings in the field of non-linear wave analysis and stochastic integrable systems, I would like to suggest the inclusion of the following recent studies:
Kai, Y., & Yin, Z. (2021). On the Gaussian traveling wave solution to a special kind of Schrödinger equation with logarithmic nonlinearity. Modern Physics Letters B, 36(02), 2150543. <https://doi.org/10.1142/S0217984921505436>
Kai, Y., Chen, S., Zhang, K., & Yin, Z. (2022). Exact solutions and dynamic properties of a nonlinear fourth-order time-fractional partial differential equation. Waves in Random and Complex Media. <https://doi.org/10.1080/17455030.2022.2044541>
Zhu, C., Al-Dossari, M., Rezapour, S., & Gunay, B. (2024). On the exact soliton solutions and different wave structures to the (2+1) dimensional Chaffee–Infante equation. Results in Physics, 57, 107431. <https://doi.org/10.1016/j.rinp.2024.107431>
Guo, S., & Wang, S. (2024). Twisted relative Rota-Baxter operators on Leibniz conformal algebras. Communications in Algebra, 52(9), 3946-3959. <https://doi.org/10.1080/00927872.2024.2337276>.

**Response: The literature review is carefully revised as suggested.**

**We are very thankful to the reviewers for their positive remarks on our proposed work, which enhanced the quality of the present work.**
